# Development and evaluation of real-time loop-mediated isothermal amplification assay for rapid detection of cystic echinococcosis

**DOI:** 10.1186/s12917-016-0809-2

**Published:** 2016-09-13

**Authors:** Mohamed E. Ahmed, Mawahib H. Eldigail, Fatima M. Elamin, Ibtisam A. Ali, Martin P. Grobusch, Imadeldin E. Aradaib

**Affiliations:** 1 Hydatid Disease Research Center, Al-Neelain Institute for Medical Research (NIMR), Al-Neelain University, Khartoum, Republic of the Sudan; 2 Molecular Biology Laboratory, Faculty of Veterinary Medicine, University of Khartoum, Khartoum, Sudan; 3 Center for Tropical Medicine and Travel Medicine, Department of Infectious Diseases, Faculty of Medicine, Amsterdam Medical Center, University of Amsterdam, Amsterdam, The Netherlands

**Keywords:** LAMP, Cystic echinococcosis, *Echinococcus granulosus*-complex, Hydatid cysts, Sudan

## Abstract

**Background:**

Cystic echinococcosis (CE) or hydatidosis, caused by the larval stage of *Echinococcus granulosus* (EG)-complex, is a neglected parasitic disease of public health importance. The disease is endemic in many African and Mediterranean countries including the Sudan. The objective of the present study was to develop and evaluate a real-time loop-mediated isothermal amplification (LAMP) assay for simple and rapid detection of CE in humans and domestic live stock in Sudan.

**Methods:**

A set of six LAMP primers, designed from the mitochondrial NADH-1 gene of EG cattle strain of genotype 5 (G5), was used as a target for LAMP assay. The assay was performed at a constant temperature (63 °C), with a real-time follow-up using a LightCycler and fluorochrome dye. Following amplification cycles in a simple water bath, LAMP products were observed for color change by naked eye and were visualized under UV light source using agarose gel electrophoresis.

**Results:**

The real-time LAMP assay identified a variety of hydatid cysts strains recovered in the Sudan, including *Echinococcus canadenses* (G6) *and Echinococcus ortleppi* (G5). Real-time LAMP positive results were detected by the presence of an amplification curve, whereas negative results were indicated by absence of fluorescence detection. Positive LAMP results appeared as a bluish-colored reaction as observed by naked eye, whereas negative LAMP results were observed as purple-colored reaction. The sensitivity studies indicated that the LAMP assay detected as little as a 10 fg of parasite DNA. There was 100 % agreement between results of the LAMP assay and our previously described nested PCR when testing 10-fold serial dilution of DNA extracted from EG-complex hydatid cyst. However, there was no cross-reactivity with other parasites including *cysticercus bovis, Fasciola gigantica*, *and Schistosoma bovis* and nucleic acid free samples.

**Conclusion:**

The developed LAMP assay would be expected to prove highly significant in epidemiological surveys of CE in developing countries or areas of resource-poor settings for both ease of use and cost.

## Background

Cystic echinococcosis (CE) in humans and susceptible animal populations is caused by the larval stage of *Echinococcus granulosus* (EG)-complex. In humans CE is considered a critical public health problem as vital organs may be severely involved. In addition, CE infection is of concern to camel producer especially in areas of endemicity, such as Tamboul region of Central Sudan [1]. Moreover, CE represents one of the neglected tropical diseases, especially in the Sub- Saharan Africa [2]. Several reports of CE have been described in humans and animals in different parts of the Sudan [3–13]. Ten distinct genotypes/strains of EG-complex designated as G1–G10 are recognized worldwide on the basis of genetic diversity. These different genotypes are associated with distinct intermediate hosts including sheep, pigs, cattle, horses, camels, goats and cervids [14–25]. So far, three EG-complex genotypes including, the sheep (G1), the cattle (G5) and the camel (G6) strains were reported in humans and livestock in the Sudan [8–10]. Epidemiological studies indicated that the camel strain (G6) represents the most prevalent genotype circulating in Sudan [26–28]. Recently, we reported, on occurrence of *Echinococcus ortleppi* (G5) in Sudanese ecotype of a dromedary camel [1]. In addition, circulating EG-complex genotypes in humans and animals is especially important in the Sudan given the large number of livestock and their importance to the national economy and rural communities. The genotypes/strains of hydatid cysts strains in areas of endemicity should be clearly defined for the purpose of epidemiological implementation and subsequent effective control measures [29–35]. In the past few years CE has been repeatedly reported as an important emerging infectious parasitic disease in Central Sudan [1, 27, 28]. It is, therefore, becoming increasingly obvious that the development of a simple and rapid molecular assay for detection of EG-complex is urgently needed particularly, in remote areas with resource-poor settings.

Molecular-based techniques are useful for detection and genotyping of EG-complex hydatid cysts. Conventional PCR assays were developed and evaluated for detection of CE [1, 36, 37]. However, most of the developed conventional PCR assays utilized a second round of nested amplification to increase the sensitivity of the assay and to confirm the identity of the primary amplified PCR product [36–38]. In addition, the PCR products may further require digestion by an endonuclease enzyme using PCR-RFLPs for genotyping of the associated EG strain. PCR-RFLPs technique is tedious, laborious and time consuming procedure [8, 16, 28, 38]. It is well documented that nested PCR is prone to error and is complicated by cross contamination due to multiple manipulations of the primary PCR products [36, 37]. To address these problems, quantitative real-time PCR (qRT-PCR) were developed instead [39, 40]. However, the developed real-time PCR assays are sophisticated techniques, which require expensive automated thermal cycler and associated PCR kits. In addition, the application of real-time PCR requires an acceptable level of training and infrastructure, which does not exist in many African countries. Recently, loop-mediated isothermal amplification (LAMP) assay has been shown to be highly accurate for the detection of echinococcosis in canine definitive hosts [41–43]. However, the previously described LAMP assays for detection of EG-complex were not monitored by real-time accelerated devices. The previously reported LAMP assays utilized sets of four LAMP primers only. In the present study, the rapidity of the LAMP assay was improved by incorporating an additional pair of loop primers (LF and LB), designed from the mitochondrial NADH-1 gene of the recently identified Sudanese strain of *E. orteleppi* [1]. In addition, the assay was performed at a constant temperature (63 °C), with a real-time follow-up using a LightCycler and fluorochrome dye. Following amplification cycles in a simple water bath, LAMP products were observed for color change by naked eye and visualized under UV light using agarose gel electrophoresis. The outer pair of LAMP primers (F3 and B3) was employed in a conventional PCR to generate a 200 bp-specific PCR product. PCR products were purified and sequenced to determine the genotype of the EG-complex hydatid cysts strain as previously described [1].

## Methods

### Collection of samples

The study was conducted during April-October, 2014. A total of hundred hydatid cysts were used in this study. Fifty hydatid cysts (*n* = 50) were collected from camel at the slaughterhouse of Tamboul, a village located at the camel producing region of Central Sudan. This slaughterhouse represents one of the major abattoirs of camel in Central Sudan. Tamboul abattoir receives animals for slaughtering from different states in Sudan including AL Gezera State, River Nile State and Khartoum State, the national capital of Sudan. Forty hydatid cysts were collected from cattle at ElKadaro slaughterhouse, Khartoum North. Ten hydatid cysts were collected from humans during surgical operations at the Khartoum Medical Teaching Hospital, Khartoum. The hydatid cysts were transferred in thermo-flasks to the Molecular Biology Laboratory at the Faculty of Veterinary Medicine, University of Khartoum, for processing and molecular detection by conventional PCR and LAMP assay. Hydatid cysts containing protoscolices and associated germinal layers were aspirated with sterile needles. The aspirates were transferred to clean sterile 50 ml tubes to which 70 % alcohol was added as preservative and stored at room temperature until used.

### DNA Extraction from hydatid cysts

The suspensions containing protoscolices and/or associated germinal layers were washed in nucleic acid free water to remove excess alcohol. Extraction of DNA from hydatid cysts was made possible using a commercially available QIAamp tissue kit (QIAGEN, Hilden, Germany) according to the manufacturer’s instructions. Briefly, 200 μl of the suspended aspirate, 20 μl of proteinase K stock solution, and 200 μl of lysing buffer were pipetted into 1.5 ml eppendorf tube. The mixture was incubated at 37 °C for 1 h and then at 70 °C for 30 min before the addition of 200 μl of absolute alcohol and mixing by vortexing. The mixture was then transferred to the QIAamp spin column placed in a clean 2 ml collection tube and centrifuged at 8000 RPM in MiniSpin centrifuge (Eppendorf, Wesseling-Berzdorf, Germany) for 1 min at room temperature. The QIAamp spin column was washed twice with 500 μl of the washing buffers by spinning for 1 min. The QIAamp spin column was placed in a clean 1.5 ml eppendorf tube and the DNA was eluted with 200 μl of double distilled water preheated at 70 °C. Maximum DNA yield was obtained by spinning at 12,000 RPM for 1 min at room temperature. From the suspended nucleic acid 5 μl was used in the PCR amplification. The extracted DNA was quantified using spectrophotometer at 260 nm wave length.

### Design of primers for LAMP assay

The primers used for LAMP amplification were designed from the nucleotide sequence of the mitochondrial NADH dehydrogenase subunit 1 (NADH 1) gene of *Echinococcus ortleppi*. The nucleotide sequence was retrieved from GenBank accession number JN637177 and aligned with the available sequences of cognate genes of other EG-complex genotypes circulating globally to identify conserved regions by using CLUSTALW software version 1.83 (DNA Data Bank of Japan; http://clustalw.ddbj.nig.ac.jp/top-e.html. A potential target region was selected from the aligned sequences. A set of six primers comprising two outer (F3 and B3), two inner (FIP and BIP), and two loop primers (LF and LB) were selected. FIP contained F1c (complementary to F1), and the F2 sequence. BIP contained the B1c sequence (complementary to B1), and the B2 sequence as shown in (Table 1). LAMP primers were designed using software PrimerExplorer V4 (http://primerexplorer.jp/elamp4.0.0/index.html; Eiken Chemical Co., Japan), as described previously by Nagamine et al. [44].

**Table 1:**
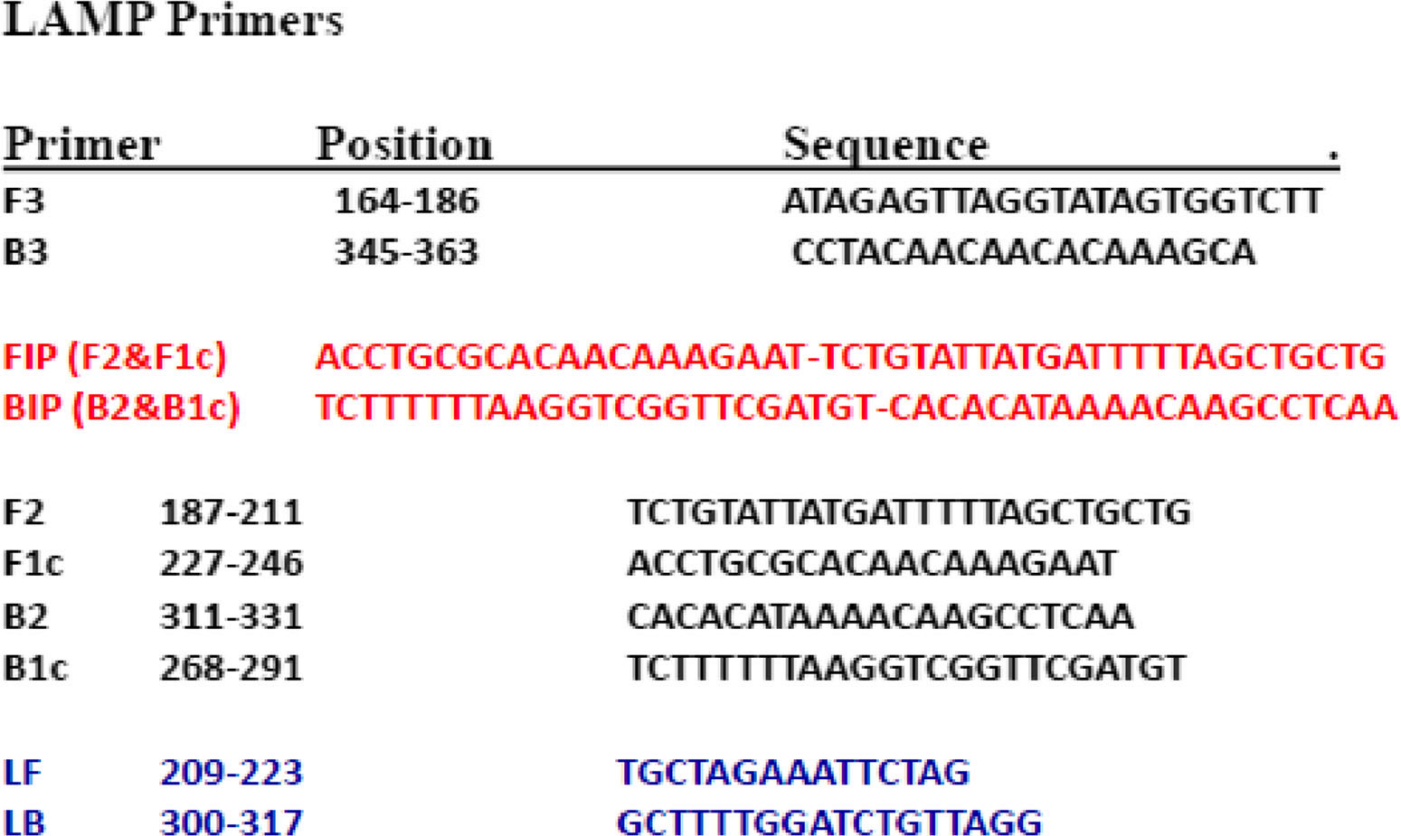
Design of LAMP primers for detection of EG-complex hydatid cysts based on the NADH 1 gene of E. ortleppi recovered from a dromedary camel in the Sudan (GenBank accession number JN637177)

### Insertion of ECO-R1 restriction sites in LAMP assay

Restriction enzyme recognition sites were inserted into each primer set. For each LAMP assay the inner primers were modified by the insertion of an EcoR1 restriction site between the F1c and F2 segments of the FIP, and the B1c and B2 segment of the BIP primer (Table 2).

**Table 2:**
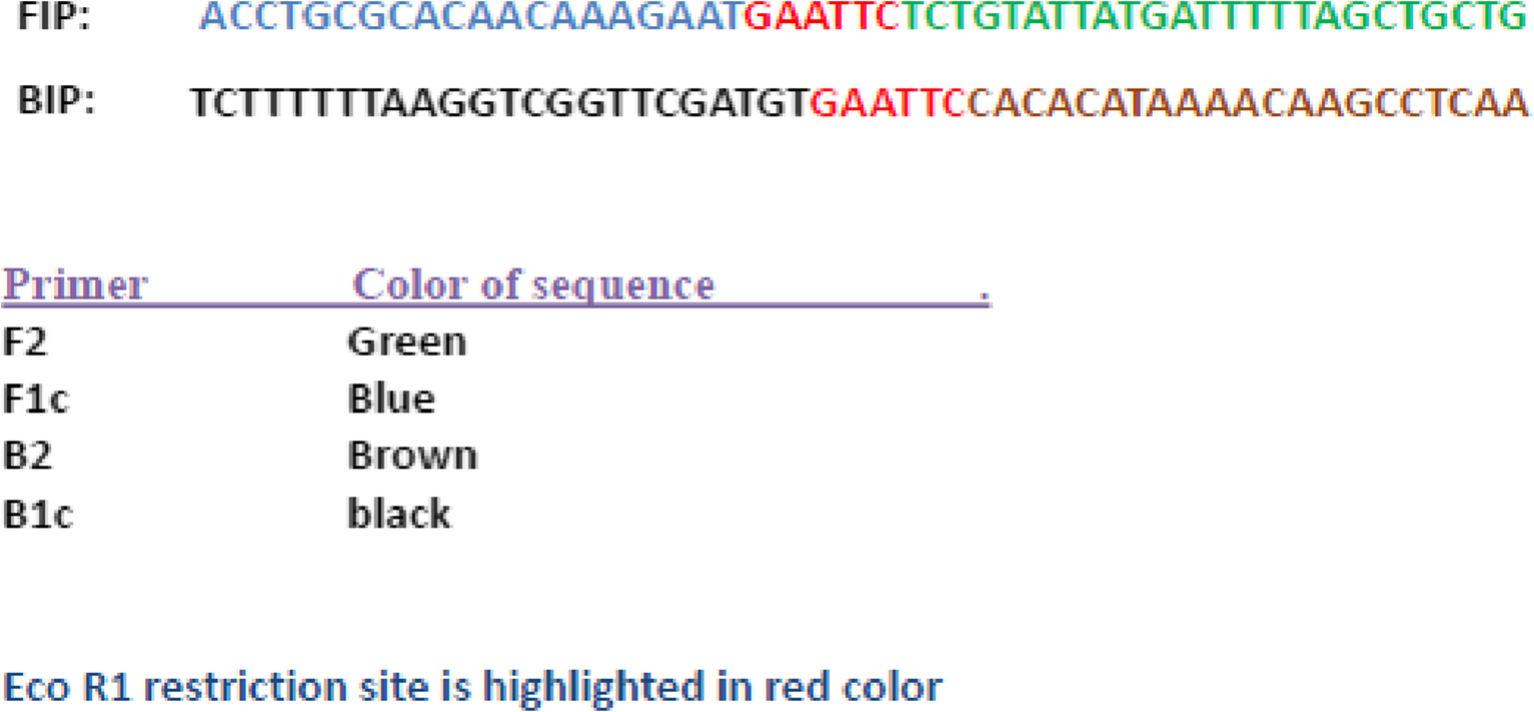
Insertion of Eco R1 restriction sites between FIP and BIP LAMP primers based on the NADH 1 gene of E. ortleppi recovered from a dromedary camel in Sudan (GenBank accession number JN637177)

### LAMP reaction conditions

The real-time LAMP assay was performed using a commercially available LAMP kit (LAMP kit, Mast Company, South Africa). The reaction condition for the LAMP assay was performed in a final volume of 25 μl per tube containing 12.5 μl 2× LAMP reaction mix. 1.0 μl of fluorochrome dye was used for real-time monitoring. 1.0 μl of detection dye was used instead for detection of color change as observed by the naked eye. 1.0 μl of Bst DNA polymerase at a concentration of 8 units per μl was used per reaction. A volume of 2.0 μl primer mixture containing (40 pmol each of the FIP and BIP primers, 20 pmol each of the LF and LB primers, and 5 pmol each of the F3 and B3 primers) was added to the LAMP reaction mix. 5.0 μl of the target DNA were added. The final volume of the LAMP reaction mix was brought to 25 μl by adding nucleic acid-free water. Positive DNA controls (EG- *ortleppi* and EG-*canadensis*) and negative DNAs controls including *cysticercus bovis, Fasciola gigantica*, *and Schistosoma bovis* and nucleic acid free samples were included in each LAMP reaction assay. The control and test DNA samples were incubated at 60–65 °C for 60 min in the LAMP assay.

### Purification and digestion of LAMP products

LAMP products generated by the modified primer mixtures containing restriction sites were purified by the QIAquick PCR Purification Kit (Qiagen, Germany) according to the manufacturer’s protocol. The products were then digested using EcoR1 enzyme (New England Biolabs, Japan) at 37 °C for 2 h.

### Real-time monitoring of LAMP assay using light thermal cycler

LAMP assay was also monitored by light thermal cycler (Rotergene Q, Australia) and a fluorochrome dye provided in the commercial LAMP kit.

### Detection of color change by the naked eye in LAMP products

Following LAMP assay in a simple water bath, Lamp products were observed by the naked eye for color change in the LAMP reaction mix using 1.0 μl of the detection dye provided in the commercial LAMP kit.

### Visualization of LAMP product by electrophoresis

LAMP products were also visualized by electrophoresis onto 2 % ethidium bromide-stained agarose gel using gel documentation system (Uvi tech, UK).

### Analysis of LAMP product with Eco R1

The generated LAMP products were digested with EC0-R1 and analyzed with a 2 % ethidium bromide-stained agarose gel electrophoresis.

### Analytical sensitivities and Specificity of the LAMP assay

The analytical sensitivities of the LAMP assay for the detection of decreasing number of hydatid cysts copies, 10-folds dilution series of the DNA standard, ranging from 10^6^ to 10^1^ per reactions, were tested in the LAMP assay. For evaluation of the specificity of the LAMP assay, DNAs extracted from other parasites including *cysticercus bovis, Fasciola gigantic*, *and Schistosoma bovis* and nucleic acid free water were used to determine the specificity of the LAMP assay for specific detection EG-complex hydatid cysts using the specific primer sets.

### Conventional PCR using LAMP outer primers (F3 and B3)

A stock buffered solution containing 150 μl 10× PCR buffer, 100 μl of 25 mM MgCl_2_, 12.5 μl of each dATP, dTTP, dGTP and dCTP at a concentration on 10 mM was prepared in 1.5 ml eppendorf tube. The primers were used at a concentration of 20 pg/μl, and double distilled water was added to bring the volume of the stock buffer solution to 1.5 ml. Each 0.5 ml PCR reaction tube contained 2 μl of the primers, 1 μl (5.0 U) of Taq DNA polymerase (QIAGEN), 5.0 μl of the target DNA and 42 μl of the stock buffered solution. The thermal cycling profiles were as follows: a 2 min initial incubation at 95 °C, followed by 40 cycles of 95 °C for 1 min, 54 °C for 30 s and 72 °C for 45 s, and a final incubation at 72 °C for 10 min. Thermal profiles were performed on a Techne TC-412 thermal cycler (Techne, Staffordshire, UK). Following amplification, 15 μl from each PCR containing amplified products were loaded onto gels of 2.0 % agarose and electrophoresed for 1 h. The gels were stained with ethidium bromide and the PCR products were easily identified using UV light source.

### Sequence analysis and genotyping

The PCR products generated by (F3 and B3) were purified using QIAquick PCR purification kit (QIAGEN) and sent to a commercial company (Macrogen, Seoul, Korea) for sequencing. Resulted sequences were edited and aligned using BioEdit software (Ibis Biosciences, Carlsbad, CA, USA). The Basic Local Alignment Search Tool (BLAST) of NCBI (National Center for Biotechnology Information, Bethesda, MD, USA) was used to confirm the identity of the generated sequences in relation to the GenBank nucleotide database. The sequences were then aligned with the corresponding regions of NADH 1 subunit genes of known genotypes from other countries to determine the genotype.

## Results

### Optimization condition and visualization of LAMP product

The optimization condition and visualization of LAMP products were determined using 10 pg of DNA extracted from Sudanese cattle strain (G5), which was incubated at a range of 60 to 65 °C. Optimum specific amplification for LAMP assay was achieved at 63 °C for 60 min.

### Detection of color change by naked eye in LAMP products

**Fig. 1:**
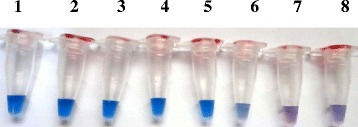
Detection by the naked eye of color change using serial dilutions of known concentration of *E. ortleppi* DNA recovered from a dromedary camel in Sudan. Blue color indicates positive LAMP result whereas purple color indicates negative LAMP result. Tube 1–8: 10-fold serial dilutions of 1.0 ng,100 pg, 10 pg, 1 pg, 100 fg, 10 fg, 1.0 fg, and DNA-free sample (negative control), respectively

Positive LAMP products were identified by detection of development of blue color in the LAMP reaction mix where as the negative samples appeared purple in color using the intercalating detection dye provided in the kit (Fig. 1).

### Real-time monitoring of LAMP assay

**Fig. 2:**
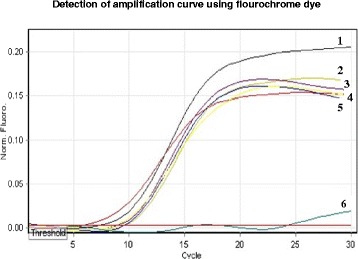
Real-time monitoring of LAMP assay using lightCycler and a fluorochrome dye. The detection of amplification curves using 1.0 pg DNA from hydatid cysts strains recovered from different animal species. Curve 1: hydatid cyst of cattle origin; curve 2–4: hydatid cyst of camel origin; curve 5: Hydatid cyst of human origin; curve 6: negative control

LAMP assay was also monitored by light thermal cycler (Rotergene Q, Australia) and a fluorochrome dye for presence of amplification curve. Positive LAMP result was indicated by the presence of amplification curve where as negative result was indicated by absence of fluorescence detection. Real-time monitoring of LAMP reaction with light thermal cycler and the fluorochrome dye provided faster results compared with the naked eye observation, where positive results could be obtained as early as 10–15 min (Fig. 2).

### Analytical sensitivity of the LAMP assay

**Fig. 3:**
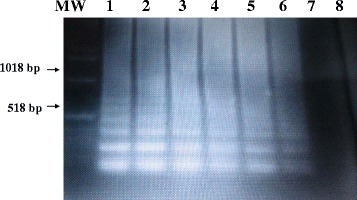
Sensitivities of the LAMP assay for detection of EG-complex hydatid cyst using ethidium bromide-stained agarose gel electrophoresis. The LAMP assay was performed with serial dilutions of known concentration of *E. ortleppi* DNA recovered from a dromedary camel in Sudan.. Lane MW: molecular weight marker; Lane 1–7: 10-fold serial dilutions of 100 pg, 10 pg, 1 pg, 100 fg, 10 fg, 1.0 fg, of parasite DNA, respectively. Lane 8: nucleic acid-free sample (negative control)

All hydatid cyst samples employed in this study were found positive in the described LAMP assay. The sensitivity of the LAMP assay was determined by testing 10-fold serial dilutions of DNA extracted from *E. ortleppi* recovered from a dromedary camel. The LAMP products were visualized by ethidium bromide-stained agarose gel electrophoresis, which produced the typical ladder-like pattern with UV irradiation. The LAMP assay has a detection limit, which span over 6 logs. High levels of analytical sensitivity were demonstrated by measuring decreasing numbers of DNA copies. The LAMP assays had 100 % sensitivity in detecting ≥ 1.0 pg of Parasite DNA (Fig. 3).

### Visusalization of LAMP product from Sudanese EG-complex genotypes

**Fig. 4:**
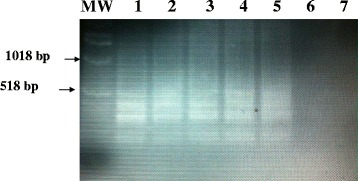
Visualization of Lamp products from fresh and archived hydatid cyst samples onto 2 % agarose gel using simple water bath. Lanes MW: Molecular marker; Lane 1: fresh sample of hydatid cyst of cattle origin; Lane 2: fresh sample of hydatid cyst camel origin; Lane 3–4: archived sample of hydatid cyst of camel origin; Lane 5–6: archived sample of hydatid cyst of human origin; Lane 7: nucleic acid-free water

Using a simple water bath set at 63 °C, and 1.0 pg of hydatid cyst DNA target, the LAMP product was detected from fresh and archive samples of EG-complex, including cattle strain (G5) and camel strain (G6) using ethidium bromide-stained agarose gel electrophoresis (Fig. 4).

### Specificity of LAMP assay

**Fig. 5:**
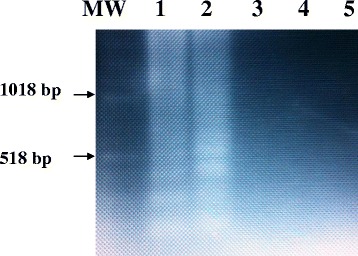
Specificity of the LAMP primers for the detection of EG-complex using *E. ortleppi* DNA recovered from hydatid cyst of a dromedary camel in the Sudan and analyzed in a 2 % agarose gel. Lanes MW: Molecular marker; Lane 1: 1.0 pg *E. ortleppi (G5)* DNA (positive control); Lane 2: 1.0 pg *E. canadensis (G6)* DNA (positive control); Lane 3: *cysticercus bovis*: Lane 4: *Fasciola gigantic*; Lane 5: *Schistosoma bovis*

The specificity studies for the LAMP assay indicated that there were no amplification products when using the specific LAMP primer set with DNA extracted from other parasites including *cysticercus bovis, Fasciola gigantica*, *and Schistosoma bovis* and nucleic acid free samples (Fig. 5).

### Digestion of LAMP product with Eco R1

**Fig. 6:**
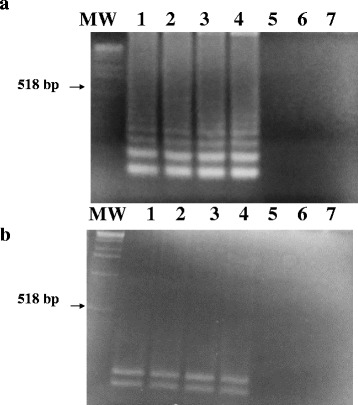
Restriction enzyme digestion of the LAMP products from hydatid cyst strains. **a** Visualization of the LAMP products from hydatid cyst strains. Lane MW: molecular weight marker; lanes 1and 2: 1.0 pg DNA from *E. ortleppi (G5)* DNA; Lane3 and 4: 1.0 pg DNA from *E. canadensis (G6)* DNA: Lane 5: *cysticercus bovis*: Lane 6: *Fasciola gigantica*; Lane 7: *Schistosoma bovis.* **b** Visualization of the restriction patterns of the digested LAMP products using Eco R1 restriction enzyme for the above gel

The specificity of the LAMP assay was further confirmed by digestion of the LAMP product with Eco-R1 restriction enzyme, which resulted in the predicted amplified products as shown in (Fig. 6).

### Conventional PCR using LAMP outer primers (F3 and B3)

**Fig. 7:**
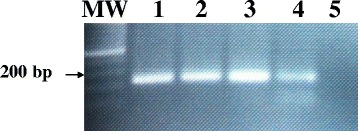
Specificity of the LAMP outer primers (F3 and B3) for amplification of the Sudanese strains of EG-complex using conventional PCR. Visualization of the 200-bp specific DNA PCR products on ethidium bromide-stained agarose gels. Lane MW: molecular weight marker; lanes 1–2: 1.0 pg *E.ortleppi* (G5) DNA (positive control); Lane 3–4: 1.0 pg *E.canadensis* (G6) DNA; Lane 5: nucleic acid-free water

The conventional PCR, using LAMP outer pair of primers (F3 and B3), resulted in amplification of a specific 200-bp PCR products. The specific PCR products were detected from 1.0 pg DNA extracted from all Sudanese genotypes of hydatid cyst including G5 and G6 strains. However, no amplification products were obtained from *cysticercus bovis, Fasciola gigantic*, *and Schistosoma bovis* and nucleic acid free samples (Fig. 7).

### Sequence analysis and genotyping

The PCR products generated by (F3 and B3) were purified using QIAquick PCR purification kit (QIAGEN). Resulted sequences were edited and aligned using BioEdit software (Ibis Biosciences, Carlsbad, CA, USA). The Basic Local Alignment Search Tool (BLAST) of NCBI (National Center for Biotechnology Information, Bethesda, MD, USA) confirmed the identity of the generated sequences and the genotypes of all hydatid cyst used in this study were confirmed as G5 or G6 strains.

## Discussion

Cystic hydatidosis is a zoonotic parasitic disease affecting both humans and livestock and has a cosmopolitan distribution [14–24]. Accumulated reports indicated that various livestock are susceptible to hydatid infection in Sudan, with particularly high prevalence in the dromedary camels [3–13, 26–28, 38, 45–47]. Early detection and genotyping of cystic echinococcosis (CE), commonly known as hydatidosis, would be advantageous in a variety of circumstances including control of the disease and subsequent prevention of spread of the infection. Rapid detection of emerging zoonotic parasitic disease, such as CE, is especially important in the Sudan given the large numbers of livestock in the country, and their importance to the economy and rural communities [45–47]. In the present investigation, we developed and evaluated a real-time and conventional LAMP assay for simple and rapid detection of fresh and archive samples of hydatid cysts using a set of six LAMP primers. The development and evaluation of a one-step, single-tube, real-time accelerated loop-mediated isothermal amplification (LAMP) for the detection of CE in humans and domestic live stock in Sudan is a simple and rapid procedure. The assay was performed at a constant temperature (63 °C), with a real-time follow-up using a LightCycler and a fluorochrome dye. The assay was highly sensitive and comparable to real-time PCR, with a detection limit of 10.0 fg of parasite DNA [40]. However, the real-time LAMP assay was much faster and generates results within 10–15 min for most employed samples. In addition to real-time detection, positive LAMP results were indicated by color change in the LAMP reaction mixed. Observation of LAMP amplified products for color change by naked eye or visualization of the products using agarose gel electrophoresis would be appropriate for most laboratory settings in developing countries [41–43]. The LAMP assay was performed under isothermal conditions and no special apparatus was needed, which makes the assay more economical and practical than real-time PCR assays. In fact, a number of PCR assays for detection of CE were described [36–39]. Together with the present study, the described LAMP assay should facilitate rapid detection and genotyping of hydatid cyst strains in a resource-poor setting in the tropics. In the present study, the potential of LAMP assay for rapid and accurate detection of CE was investigated, on a practical scale for the first time in Sudan. The LAMP assay provides high levels of diagnostic sensitivity and specificity when testing a variety of cysts sampled from human and domestic live stock. Using the detection dye, processing, extraction of parasite DNA and application of LAMP assay could be completed in approximately 90 min after arrival of the samples in the laboratory. However, the estimated time for real-time detection of a LAMP positive result was significantly reduced when using LightCycler and fluorochrome dye. Positive LAMP results could be monitored as early as 5–10 min before completion of the cycles, which last for 60 min. In addition, an important practical advantage of the LAMP technique is that it utilizes simple and relatively inexpensive equipment, such as a simple water bath or heat block, which renders the assay promising for use in rural and remote areas with resource-poor settings. Moreover, only basic molecular and technical skills are required for performance of the LAMP assay procedure, and interpretation of the results may be as simple as a visual evaluation of color change in the reaction mix.

The sensitivity studies indicated that the LAMP detected 10.0 fg of parasite DNA as indicated by color change in the reaction mix, which is most likely the way it would be read in a resource-poor setting. Using agarose gel electrophoresis, the LAMP assay detected as little as 10 fg of parasite DNA. Our results illustrate that the sensitivities of the developed LAMP assay and our previously described nested RT-PCR assays are in 100 % agreement and both assays exhibit high levels of analytical sensitivity [36]. However, nested PCR is prone to error and is complicated by cross reaction due to multiple manipulations of PCR products.

The specificity studies indicated that no cross reactivity was detected with 1.0 pg DNA from *cysticercus bovis, Fasciola gigantica*, *and Schistosoma bovis* nucleic acid free samples under the same stringency condition described in this study. In the present study the LAMP assay was evaluated for detection of the Sudanese genotypes of EG-complex hydatid cysts. This study does not deal with sensitivity/specificity testing on a large practical scale but rather constitutes a principle for application of LAMP assay for diagnosis of CE.

Since the LAMP primers were designed based on multiple sequence alignment of several published sequences of the NADH 1 gene, using BioEidit software (Carlsbad, CA, USA), and were selected from a highly conserved fragment of the gene, they would be expected to amplify DNA from all genotypes of EG-complex hydatid cyst strains circulating globally. However, DNAs from other genotypes of hydatid cyst strains were not available in the Sudan to be included in this LAMP assay. Therefore, additional research would be necessary to confirm this assumption. The described LAMP assay can have great potential in developing African countries, such as Sudan, where the disease is endemic and equipment and expert technical staff is scarce. The cost of the described LAMP assay should be around that of the conventional PCR assay, if not less expensive. In fact, the LAMP assay utilizes Bst enzyme for amplification of the target sequence. However, Taq DNA polymerase enzyme is required for conventional or real time PCR amplification, which is more expensive than Bst enzyme. The described real-time LAMP assay could very easily be adjusted for coprodiagnosis of EG-complex eggs in fecal samples from infected canines. The role of this LAMP assay in coprodiagnosis and its application in epidemiological studies and disease control programs should be promising and highly significant. It is worth mentioning that conventional parasitological method could be useful for diagnosis of hydatid cyst under the microscope but has no significance in genotyping of the parasite. However, the LAMP assay, described in this study, could be employed for simultaneous detection and genotyping of cysts recovered from infected livestock. It is well documented that different genotypes exhibit different pathological consequences, transmission profiles, and sensitivity to chemotherapeutic agents. These biological variations should be considered in developing vaccines, diagnostic kits and pharmacological therapies for control of CE. In the present study, genotyping of the hydatid cyst strains was made possible by using the outer pair of LAMP primers (F3 and B3) in a conventional PCR assay and subsequent sequencing of the specific PCR product. The genotypes of all strains of hydatid cyst used in this study were confirmed as Echinococcus canadensis (G6) or Echinococcus ortleppi (G5) using ClustalX (http://www.clustal.org/) as described previously [1].

## Conclusion

In conclusion, the LAMP assay, described in this study, could be used for simple and rapid detection and genotyping of EG-complex hydatid cysts strains. There was 100 % agreement between results of the LAMP and our previously described nested RT-PCR when testing 10-fold serial dilution of parasite DNA. The LAMP assay provides very high levels of diagnostic sensitivity and specificity when testing a variety of archived hydatid cysts sampled from human or susceptible animal populations. Real-time monitoring of the LAMP assay using LightCycler and fluorochrome dye enhanced the rapidity of the assay and a positive result could be obtained as early as 10–15 min post amplification reaction. The performance of the LAMP assay under isothermal conditions without the need of special apparatus, and visualization of results by the naked eye, makes the assay more economical and practical in remote areas or resource-poor settings. Partial sequences produced by LAMP outer primers (F3 and B3) could be targeted for sequencing and subsequent identification of the genotype of the hydatid cyst genotype/strain.
